# Study on the Effect of Streets’ Space Forms on Campus Microclimate in the Severe Cold Region of China—Case Study of a University Campus in Daqing City

**DOI:** 10.3390/ijerph17228389

**Published:** 2020-11-12

**Authors:** Hong Jin, Liang Qiao, Peng Cui

**Affiliations:** 1 Key Laboratory of Cold Region Urban and Rural Human Settlement Environment Science and Technology, Ministry of Industry and Information Technology, School of Architecture, Harbin Institute of Technology, Harbin 150006, China; jinhong@hit.edu.cn (H.J.); 14b334004@hit.edu.cn (P.C.); 2 School of Civil Engineering and Architecture, Northeast Petroleum University, Daqing 163318, China; 3 School of Landscape, Northeast Forestry University, Harbin 150040, China

**Keywords:** server cold area, college campus, microclimate, street morphology, L/C (plane opening rate)

## Abstract

In urban areas, local microclimate is influenced by architectural forms, which will in turn affect human comfort. Taking Daqing as an example, this article studies the microclimate of a university campus in the severe cold area in China. Based on the space features of the streets, we categorize the streets into three types: open type, semi-open type, and street-entry type. Through analysis, this article researches microclimates of the three kinds of streets, the influence of building heating on the surrounding thermal environment, the relationship between streets’ morphology features and microclimate and human comfort (physiological equivalent temperature, PET). By study and analysis, we have the following findings: for open-type streets, the average globe temperatures of streets with different orientations can reach 1.3 °C in winter because of the influence of sidewalk trees. For semi-open-type streets, streets temperature is under the influence of the locating directions of buildings. The maximum air temperature difference among streets with different building arrangements reaches 2.1 °C in winter. For street-entry-type streets, the height–width ratios and orientations of streets are related to the continuity degree of the street interfaces. The building interface acts as a heating element and affect the surrounding thermal environment by heat convection and heat radiation. Analysis demonstrates that heat convection has a more obvious effect on rising surrounding temperature than heat radiation. Buildings with higher heat radiation witness higher globe temperature. For street-entry-type streets and semi-open-type streets, the SVF (sky view factor) and L/C (plane opening rate) of streets are negatively correlated with temperature and PET, but positively correlated with wind speed. If the SVF increases 0.1, the air temperature will reduce 0.1 °C, the wind speed will increase 0.19 m/s, and the PET will reduce 0.7 °C.

## 1. Introduction

In recent years, people’s living environment has been affected by the acceleration of urbanization, the rapid expansion of cities, and the continuous deterioration of the city’s microclimate. At the same time, the rapid development of cities has increased energy consumption in urban buildings [1]. As an important part of a city, college campuses are both interconnected with and relatively independent of the urban microclimate. Undertaking multiple functions, college campuses have a very complex space form and low plot ratio. Accordingly, college campuses have unique street systems and open space design. On a microscopic level, the geometry of streets and the setting of open spaces affect their micro climatic elements, such as sunlight, windspeed, humidity, temperature, and the like [2]. As a result, college campuses have distinct microclimate features.

Among various factors, Mohajeri [3] found that the orientation of street imposes the greatest influence on the amount of solar radiation available on the street. The air temperature of the street depends on the average radiation temperature, and the average radiation temperature is directly affected by solar radiation. The larger the area of the street receiving solar radiation, the higher the average radiation temperature is [4]. Chatzidimitriou’s [5] research indicates that NE-SW oriented streets have higher temperature in winter. At the same time, wind speed differences among streets of different orientations can reach 2.7 m/s. According to Sözen’s [6] study, the average wind speed of N–S (North–South) oriented street is about 3.7 m/s faster than that of the E–W (East–West) oriented street, and is about 1.1m/s faster than that of the NE–SW oriented street. Considering thermal comfort and solar radiation in winter, NE–SW oriented streets and NW–SE oriented streets are the best choice [7,8]. The studies mentioned above suggest that streets’ orientations have a big influence on streets’ microclimate. However, the streets under studies are mostly continuous and complete. However, for streets with more open spaces and less continuous form, it seems that researchers have reached a few conclusions.

The trees, by affecting sun exposure and evaporating, will improve the comfort level of the surrounding environment, especially in tropical and subtropical cities [9,10]. The shadow effect of trees will reduce the mean radiant temperature (MRT) of streets [11]. Morakinyo [12] has studied the effect of trees on the microclimate of urban streets canyon in Hong Kong. According to the study, parameters of trees (such as leaf area index, tree height, and tree trunk height) influence thermal comfort during daytime and nighttime. The study recommends that tall and big trees of low-density are more preferable for deep canyons, while the shallow canyons and open areas could choose the opposite. These studies on trees mainly focus on trees’ shadow effect in summer but pays little attention on how deciduous trees affect the thermal environment in winter.

The SVF (sky view factor) indicates the capability of urban space to receive solar radiation. The value of SVF is determined by architecture and greening [13]. Areas with lower SVF values generally have lower temperatures during the day because they receive a lesser amount of solar radiation during the daytime [14,15,16,17,18,19]. There exists a significant linear relationship between SVF and annual global irradiance in all orientations (*R^2^* > 0.8), and the solar altitude angle has a strong effect of solar radiation [20]. SVF has a limited impact on the outdoor air temperature in summer but has a big impact on the globe temperature [21]. At the same time, SVF also has a great influence on wind speed. Comparing the results of various studies, we find that, under different climates and environmental conditions, the spatial forms of different spaces have different impacts on the microclimate [22]. Pearlmutter [23], Bourbia [24], and Ali-Toudert [25] have successively studied the relationship between the street morphological indicators (such as height–width ratio and street orientation) and the street thermal environment. The studies suggest that streets temperature is closely related to the height–width ratio and orientations of streets. Cui [26,27] establishes the quantitative equation between morphological parameters and air temperature in Singapore, and finds that, when the radius is 50 m, morphological parameters have the biggest influence on temperature. 

Space forms of campus affect its microclimate [28]. The forms of buildings have a significant influence on sunshine duration, average radiation temperature, and wind environment [29]. Based on the study of campus microclimate in a severe cold region, we reduce the duration of uncomfortable time by 25% and reduce building’s heat release by 5% if we design the outdoor space forms properly by considering outdoor comfort and building heat release in summer [30]. As an important part of the urban blocks, college campuses have a more diverse space form. Generally speaking, the plot ratio of college campuses is low and their spaces are more open. The students spend a long time in the outdoor environment every day. Thus, the comfort level of the outdoor environment deserves more attention. Accordingly, the purposes of this study are as follows:

1. Categorize street forms into different types based on analysis, and study their respective microclimate features;
2. Study the influence of buildings’ interfaces on the surrounding thermal environment;
3. Establish quantitative equations between street forms and microclimate.

## 2. Research Methods

### 2.1. Study Area and Monitoring Sites

**Figure 1:**
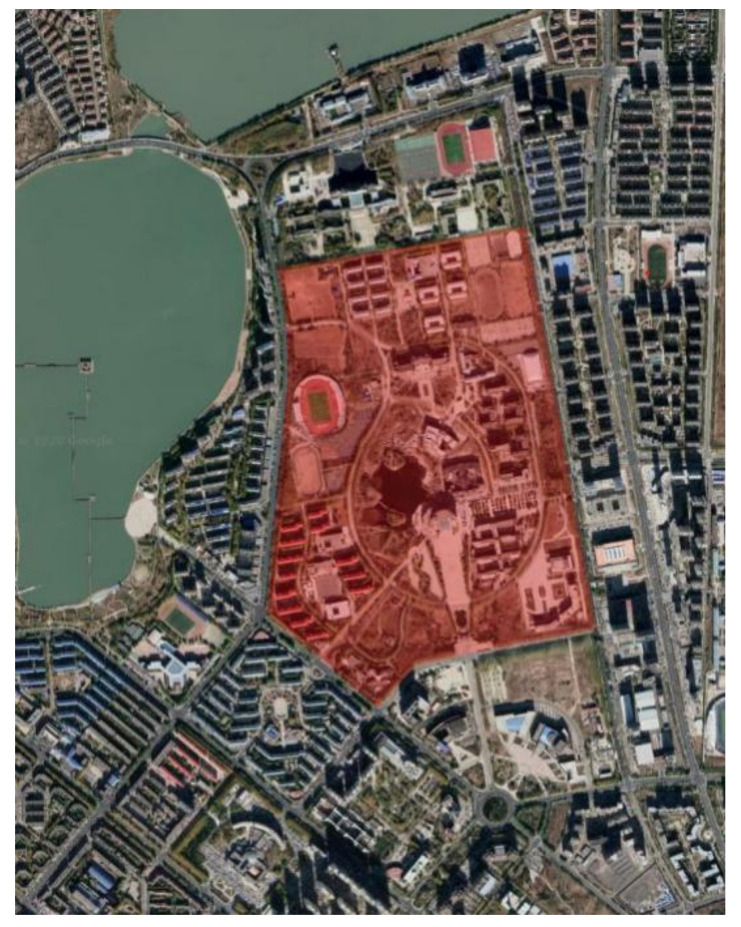
The campus and surrounding layout.

Daqing, a typical city in the severe cold area, is selected for this study. The climate of Daqing is shown in Table 1. Our tests are carried out in winter (27 December 2018, 14 January 2019, and 16 January 2019). The monitoring site is the campus of Northeast Petroleum University, which is located in the center area of Daqing. It covers an area of 1.503 million square meters. The teaching and administrative areas cover 600,000 square meters, and the indoor and outdoor sports venues cover 157,000 square meters. On the campus, the east side and the west side are mainly covered by high-rise residence, the south side has many public buildings, and the north side is near multi-story teaching buildings. Layout and forms of the campus are shown in Figure 1.

**Figure 2:**
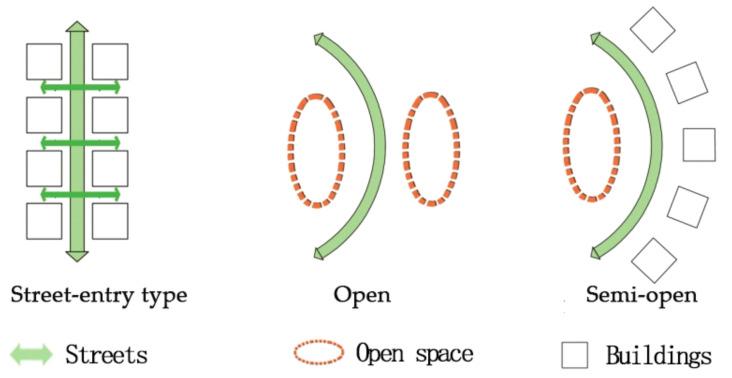
Space types of campus streets.

In severe cold regions of China, college campuses are mainly located in the center part of urban areas. The functions of campus determine the uniqueness of their planning layout. Therefore, the texture and space form of campus are quite different from other places. The outdoor environment of the campus is quite spacious and the plot ration of the campus is relatively low. Based on the different space forms of campus streets, the streets may be categorized into three types: open type, semi-open type, and street-entry type. The open type has open space on both sides, the semi-open type has buildings on one side and open space on the other side, and the street-entry type has buildings on both sides. Forms of the three kinds of streets refer to Figure 2.

### 2.2. Test Instruments and Methods

**Figure 3:**
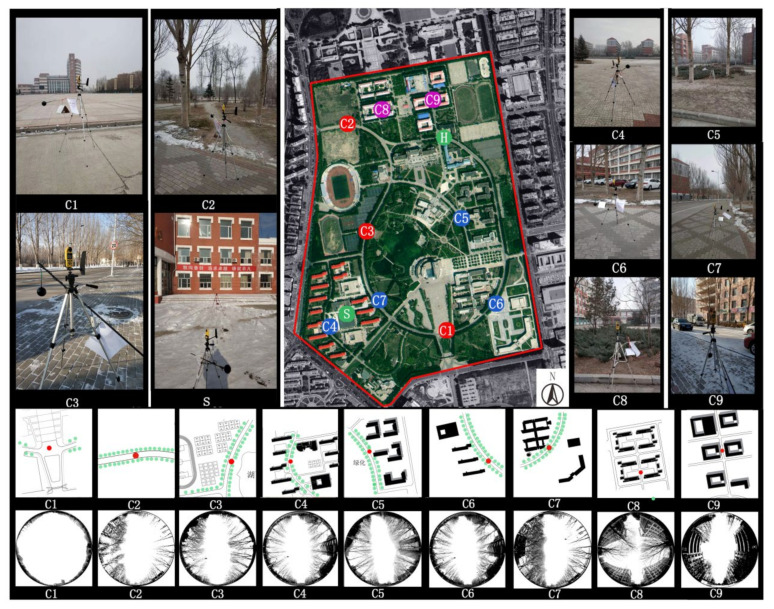
Arrangement of monitoring sites (C1–C9) and fisheye lens photos. Each color represents a type of street for easy contrast.

The test time and the climatic conditions are shown in Table 2. The monitoring sites may be divided into two groups. The first group includes nine fixed monitoring sites, labeled as C1, C2, C3, C4, C5, C6, C7, C8, and C9. This group is used for studying the microclimates of different street types. The second group of monitoring sites are set along the vertical direction of building walls, labeled as S1, S2, S3, and S4. This group is used for studying the influence of the heated building walls on the thermal environment of the street space. Specific arrangement of the monitoring sites is shown in Figure 3.

**Figure 4:**
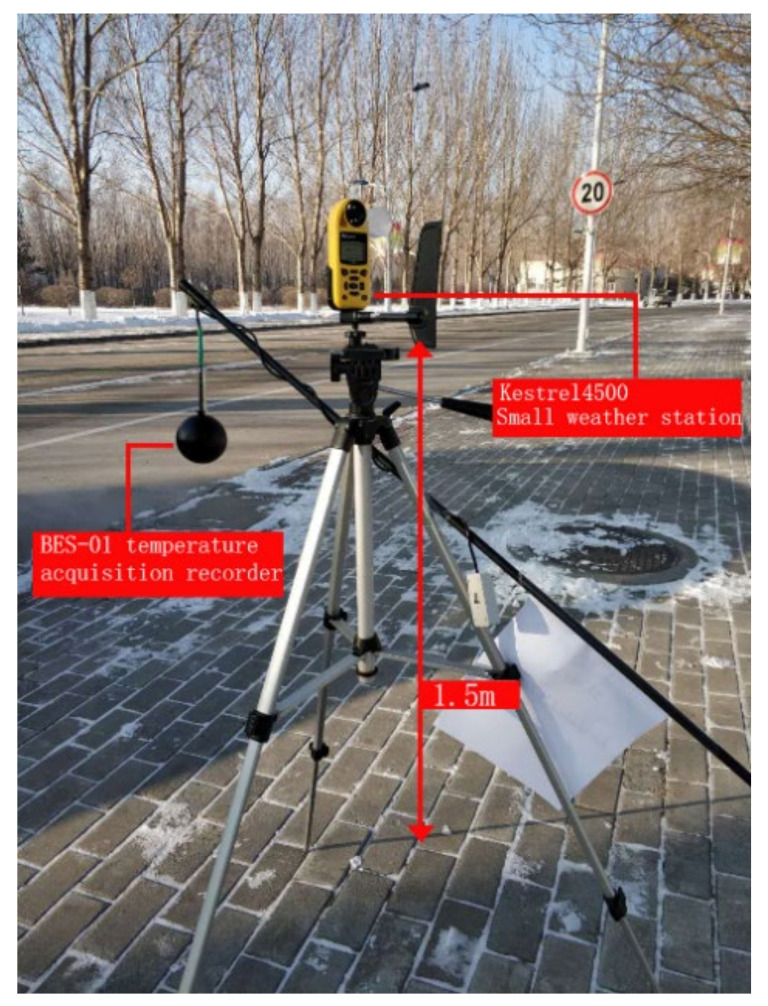
Weather station structure.

The test comprehensively considers the impacts of air temperature, relative humidity, wind and solar radiation on the campus thermal environment. The BES-01 (Institute of building energy saving technology of Harbin Institute of Technology, Harbin, China), temperature recorder and the Kestrel 4500 (Nielsen-Kellerma, Boothwyn, PA, USA) small-size weather station are used to record data such as globe temperature, air temperature and wind speed. Technical parameters of the instruments are listed in Table 3. Before the test, the instruments have been calibrated multiple times. As the research focuses on the thermal environment at the pedestrian height, the test instruments are fixed on tripods at a height of 1.5 m from the ground (see Figure 4). The test data are recorded at an interval of 1 min. Physiological equivalent temperature (PET) is an index used for the biometeorological assessment of the thermal environment. In this study, we use RayMan’s model to calculate PET [31].

### 2.3. Calculation of the Campus Morphology Parameters

Temperature and wind environment of the urban microclimate are directly related to the geometry forms of the urban street space [32,33]. Closure degree is a kind of variable describing the geometry forms of space. According to the studies we mentioned in the introduction part, closure degree of street is an important factor influencing microclimate of streets. In this study, we choose three parameters for testing, including plane opening rate (L/C), sky view factor (SVF), and build-to-line ratio, all of which belong to indicators of closure degree. The plane opening rate (L/C) refers to the ratio of the perimeter of the continuous interface surrounding the site space to the width of the space opening. Sky view factors (SVF) refers to the area ratio of the sky visible area at a height of 1.5 m to the hemispherical sky in the building site. Build-to-line ratio refers to the percentage of the length formed by building walls and the street length. Build-to-line ratio is a parameter for street continuity. To study SVF, we take pictures of each monitoring sites with Canon fisheye lenses (EF8–15 mm, f/4L, Ultra Sonic Moto, Canon, Tokyo, Japan), and calculate SVF value by Rayman 1.2 (Albert-Ludwigs-Universität Freiburg, Freiburg, Germany) (see Figure 1). In terms of L/C, the scope of influence is set to 200 m, since the school building blocks and the open spaces are relatively large. Field research and Google Maps are combined in our study, and the L/C is calculated by Auto CAD (Version 2012-education, Autodesk, San Rafael, CA, USA) [34]. Build-to-line ratio is also calculated through this method. Features of the monitoring sites’ outer space are shown in Table 4.

## 3. Results and Analysis

### 3.1. Microclimate Features of Different Street Forms

#### 3.1.1. Microclimate Features of Open-Type Streets

Open-type streets have two different kinds–one kind has trees on both sides (C2 and C3), and the other kind has open space on both sides (C1). C2 and C3 have similar SVF and L/C, but they differ in street orientations: C2’s orientation is EW-N5°, and C3’s orientation is SN-E15°. By comparing the microclimatic parameters of C2 and C3 in winter, we find out the effects of street orientation on microclimate. 

**Figure 5:**
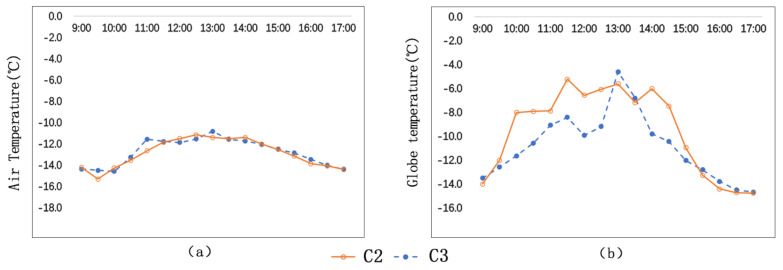
Comparison of air temperatures and globe temperatures of C2 and C3: (**a**) Ta; (**b**) Tg.

The test results (Figure 5a) show that C2 and C3 have similar air temperature (Ta) with slight differences. At C3, the mean value of air temperature is −12.7 °C; and, at C2, the mean value of air temperature is −12.9 °C. C3’s mean air temperature is 0.2 °C higher than that of C2. The air temperature peak of C2 occurs at 12:30 p.m., and that of C3 occurs at 1:00 p.m. At 11:00 a.m., the air temperatures of C2 and C3 have a difference of 1.1 °C, the biggest difference during the day. 

The test results (Figure 5b) show that the globe temperature (Tg) of C2 is significantly higher than that of C3, and the globe temperature of C2 witnesses a bigger temperature fluctuation. The mean globe temperature of C2 is −9.5 °C, and the mean globe temperature of C3 is −10.8 °C. Their difference is 1.3 °C. Their globe temperature peaks occur at different times: C2’s peak temperature is −5.2 °C, occurring at 11:30, while C3’s peak temperature is −4.6 °C, occurring at 1:00 p.m. At 2:00 p.m., the globe temperatures of C2 is 3.8 °C higher than that of C3, the biggest difference during the day. 

By analysis, we can find that streets of different orientations have different temperature features. The reason is that solar altitude in winter is low, and that street trees cause different degrees of sun blockage due to different street orientations. Street orientations have a bigger effect on globe temperature (Tg) than on air temperature (Ta). The average globe temperature of EW-N5°oriented streets is 1.3 °C higher than that of SN-E15° oriented streets.

**Figure 6:**
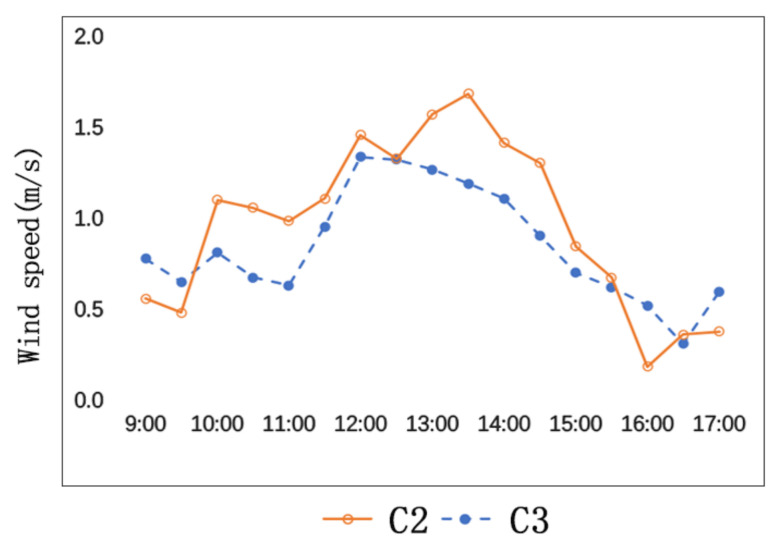
Comparison of wind speeds of C2 and C3.

As is shown in Figure 6, the average wind speed of C2 is bigger than that of C3. The wind speeds of C2 and C3 are 0.9 m/s and 1.0 m/s respectively, and their difference is 0.1 m/s. The maximum wind speed of C2 is 1.7 m/s, and the maximum wind speed of C3 is 1.3 m/s, and their difference is 0.4 m/s.

The general environments of C2 and C3 are similar, but they have different street orientations and different angles with the predominant wind direction. The angle formed by C2 and the predominant wind direction is smaller than the angle formed by C3 and the predominant wind direction. In addition, the sidewalk trees on both sides of C2 together with the predominant wind form a canyon effect, which also accelerates the wind speed.

C1 and C2 have the same orientation. On both sides of C1, there are open spaces, while, on both sides of C2, there are sidewalk trees. By comparing the microclimate differences of the two streets, we study the impact of street trees on microclimate. 

**Figure 7:**
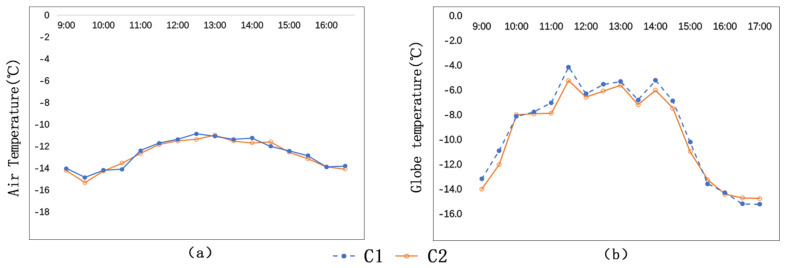
Comparison of air temperatures and globe temperatures of C1 and C2: (**a**) Ta; (**b**) Tg.

As is shown in Figure 7a, the change trends of air temperatures of the two streets are similar. The average air temperature of C1 (−12.7 °C) is 0.1 °C higher than that of C2 (−12.8 °C). The highest air temperature of C1 is −10.6 °C, which occurs at 12:30 p.m., and the highest air temperature of C2 is −11 °C, which occurs at 1:00 p.m., half an hour later than C1.

As is shown in Figure 7b, the globe temperature trends of C1 and C2 are similar as well. The average globe temperature of C1 (−9.1 °C) is higher than that of C2 (−9.5 °C). Their difference is 0.4 °C. The highest globe temperature of the two measuring sites are found at 11:30 a.m. The highest globe temperature of C1 is −4.1 °C, and the highest globe temperature of C2 is −5.2 °C. Their difference is 1.1 °C.

**Figure 8:**
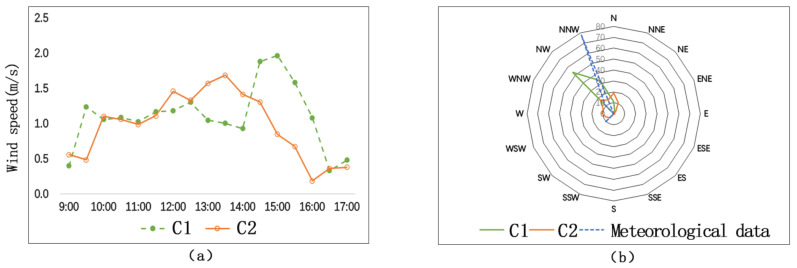
Comparison of wind speeds and wind direction frequencies of C1 and C2: (**a**) wind speeds; (**b**) wind direction frequencies.

As is shown in Figure 8a, C1 and C2 witness similar change trends in wind speed. The average wind speed of C1 is 1.1 m/s, and the average wind speed of C2 is 0.9 m/s. Their difference is 0.2 m/s. The biggest difference of wind speed between the two measuring sites is 1.1 m/s, which occurs at 3:00 p.m. The SVF of C1 (no trees) is 0.85, and the SVF of C2 (with trees) is 0.63. The lower the closure degree, the greater the wind speed. 

As is shown in Figure 8b (the wind-rose diagram during this period), the wind direction of C1 has a closer relationship with the meteorological wind direction, and the wind direction of C2 has a wider variation range. Therefore, we conclude that wind direction of the street with trees is not closely related to the predominant wind direction.

#### 3.1.2. Microclimate Features of Semi-Open-Type Streets

C4, C5, C6, and C7 belong to semi-open-type streets. Their common feature is that the street has buildings on one side and open spaces on the other. The street orientations of these sites are similar. Thus, in this part, we mainly study how building location, height–width ratio of street, SVF, and L/C influence temperature and wind speed. 

**Figure 9:**
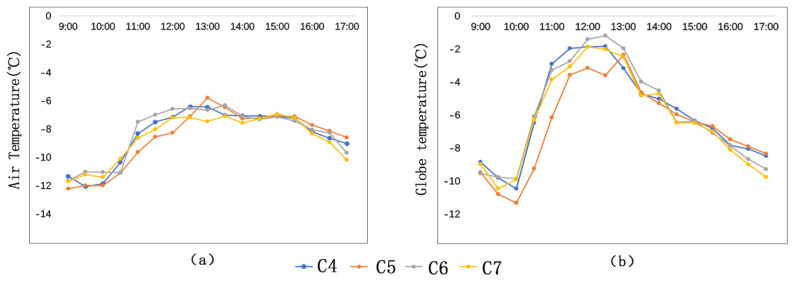
Air temperature comparison and globe temperature comparison at the semi-open-type streets: (**a**) Ta; (**b**) Tg.

Figure 9 shows that before 12:30 p.m., the air temperature (Ta) and globe temperature (Tg) comparison result of the measuring sites is C6 > C4 > C7 > C5. The maximum air temperature difference of the four measuring sites is 2.1 °C (C6 > C5), and the maximum globe temperature difference of the four measuring sites is 2.8 °C (C6 > C5).

**Figure 10:**
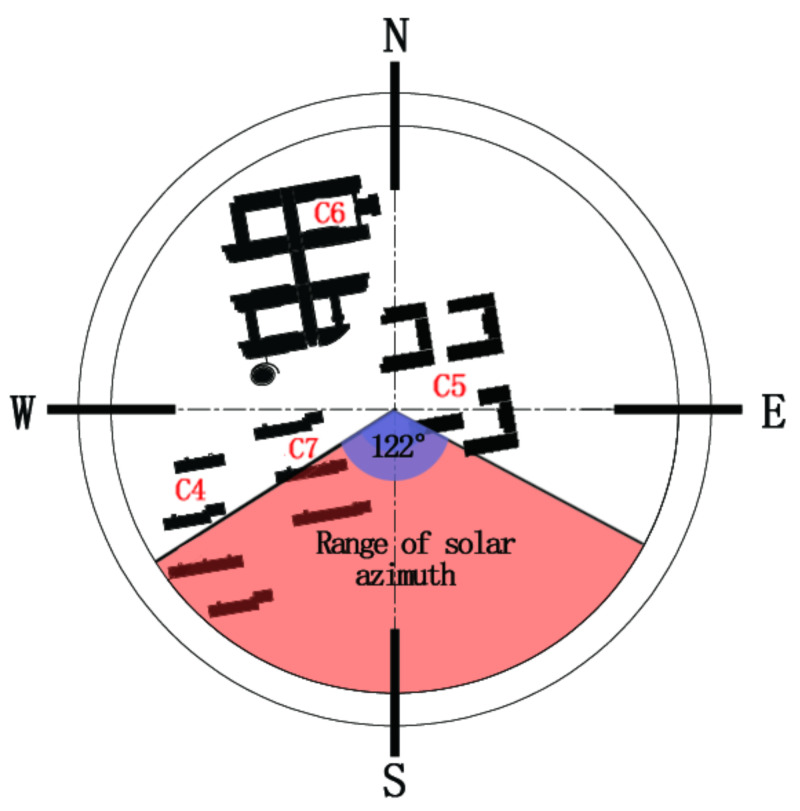
Relationship between locating directions of buildings and solar azimuth.

The reason for this result is that buildings of these streets are located in different directions and thus have different degrees of sunshine blockage (sunshine blockage degree C6 < C4 < C7 < C5). Figure 10 demonstrates the relationship between the locating directions of the buildings and the solar azimuth. At C5, the buildings are high (36 m), and the height–width ratio of the street is 3.6. These buildings block much solar radiation. As a result, C5 has the lowest temperature.

At C4 and C7, the buildings are located in similar directions. For C4 and C7, their maximum air temperature (Ta) difference is 0.8 °C (C4 > C7), and their maximum globe temperature difference is 1 °C (C4 > C7). The SVFs of C4 and C7 are 0.47 and 0.57, respectively, and the L/Cs of C4 and C7 are 0.49 and 0.77, respectively. It can be concluded that lower SVF and L/C correspond to higher temperature.

**Figure 11:**
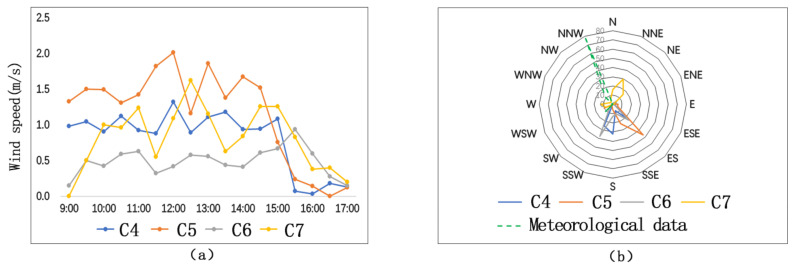
Comparison of wind directions and wind frequencies on semi-open-type streets: (**a**) wind speeds; (**b**) wind direction frequencies.

As is shown in Figure 11a, the comparison result of average wind speed of the four measuring sites is C5 (1.16 m/s) > C7 (0.82 m/s) > C4 (0.81 m/s) > C6 (0.49 m/s). Although the respective wind speeds of the four sites change frequently, their general trends of wind speed change are similar. On the measuring day, the observatory average wind speed is 3.2 m/s. Wind speeds of the four measuring sites are quite different from the average wind speed of the meteorological station. It shows that spatial form has an obvious influence on the wind environment. 

On the measuring day, the predominant wind direction is northwest wind. On the upwind direction (the northwest direction) of C6, there are continuous teaching buildings. Affected by the building group, wind speed of C6 is the lowest. On the downwind direction of C5, there is a discontinuous space interface. Interacting with the high-rise buildings and the predominant wind, the wind speed of C5 is the fastest. C4 and C7 have similar directions. At C4 and C7, there are strip-type dormitory building groups at the upwind direction, which helps to reduce the wind speed. Therefore, wind speeds of C4 and C7 are faster than that of C6, but lower than that of C5.

Figure 11b—the wind-rose-diagram—shows that wind directions of the four measuring sites are not consistent with the predominant wind of the day. Wind environments of the four measuring sites are affected by their respective spatial forms. The predominant wind of C4, influenced by the rank of building groups, changes its direction. At C6 and C7, the open areas are large, and thus wind can blow from the open area to the testing sites. At C5, the high-rise building blocks the wind flow and forms a vortex zone on windward. Thus, the wind direction is changed at C5. Therefore, on semi-open-type streets, discontinuous building interface reduces the wind speed and changes the wind speed. If there are high-rise buildings on the windward side, vortex zone will be formed, which will in turn change the wind direction and accelerate wind speed.

#### 3.1.3. Microclimate Features of Street-Entry-Type Streets

**Figure 12:**
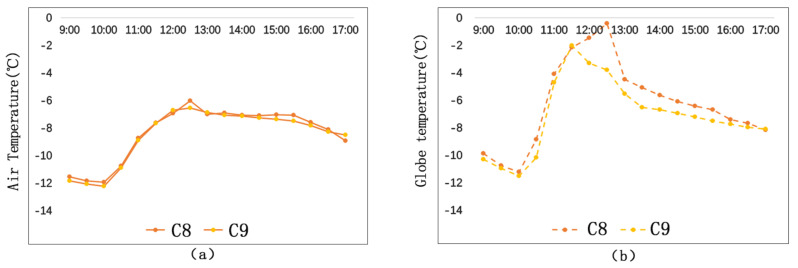
Air temperature comparison and globe temperature comparison on street-entry type streets: (**a**) Ta; (**b**) Tg.

Figure 12a shows that air temperatures of C8 and C9 are similar. Their maximum air temperature difference between C8 and C9 is 0.5 °C, occurring at 12:30 p.m.

Figure 12b shows that globe temperature of C8 is obviously higher than that of C9. The average globe temperature of C8 is 0.85 °C higher than that of C9, and their maximum globe temperature difference is 3.4 °C, also occurring at 12:30 p.m. 

With respect to street-entry-type streets, street orientation and street height–width ratio are the main factors affecting microclimate. Street orientations of C8 and C9 are the same, and their height–width ratios are also the same. The main spatial difference between C8 and C9 is the continuity of the building interfaces. Build-to-line ratio is a parameter to determine spatial continuity. The street wall facades of C8 and C9 are evenly arranged. The build-to-line ratio of C8 is 32%, and the build-to-line ratio of C9 is 62%. The larger the build-to-line ratio is, the more continuous the space is. Such kind of continuous and closed space form will block solar radiation and lower the temperature. 

**Figure 13:**
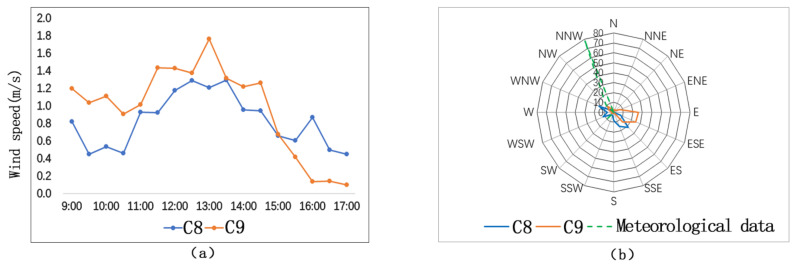
Comparison of wind speeds and wind direction frequencies on street-entry-type streets: (**a**) wind speeds; (**b**) wind direction frequencies.

From Figure 13a, we could see that the change trends of wind speed are similar at C8 and C9. The average wind speed of C9 (1.0 m/s) is 0.2 m/s higher than that of C8 (0.8 m/s), and the maximum difference between the two sites is 0.6 m/s, occurring at 4:00 p.m. The SVF and the L/C of C9 are higher than those of C8. Accordingly, we find out that wind speed is lower in a more closed space. 

Figure 13b shows that the predominant wind directions of C8 and C9 are similar, but both are different from the predominant wind direction of the day. Such difference is caused by the street orientations and geometrical forms of the two measuring sites. At the east and west sides, some parts of the buildings are built on stilts. Such architectural form interacts with a chimney effect, resulting in the change of wind direction. In conclusion, wind environment is greatly affected by the spatial forms of the streets. 

### 3.2. Influence of Building Heating Interface

**Figure 14:**
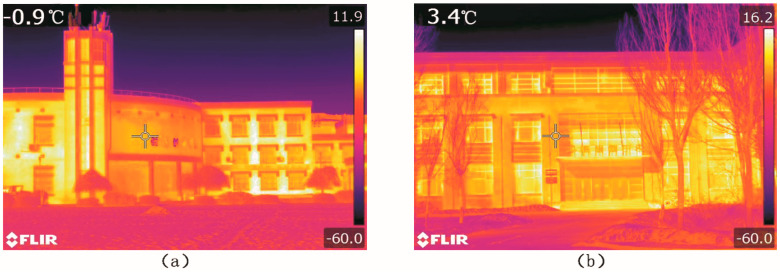
Infrared photos of buildings at measuring points S and H: (**a**) measuring points S; (**b**) measuring points H.

In severe cold regions, people feel uncomfortable in winter. The building heating in winter can be regarded as a heating source. In addition, the building walls can receive solar radiation and generate long-wave radiation, which in turn change the surrounding thermal environment. Figure 14 is an infrared photo taken at the measuring site S and site H. On the observation day, the highest temperatures of the wall surfaces are 11.9 °C and 16.2 °C, respectively, at site S and site H. Therefore, we can say that the building heating has a certain influence on the outdoor thermal environment. 

**Figure 15:**
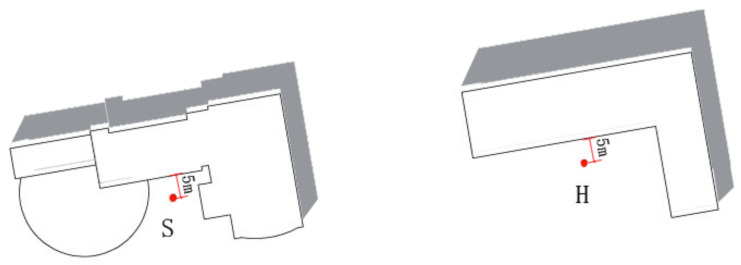
Measuring points S and H.

The measuring points S and H are located at the places 5 m from the building walls (see Figure 15). We analyze the influence of different building walls on the thermal environment.

**Figure 16:**
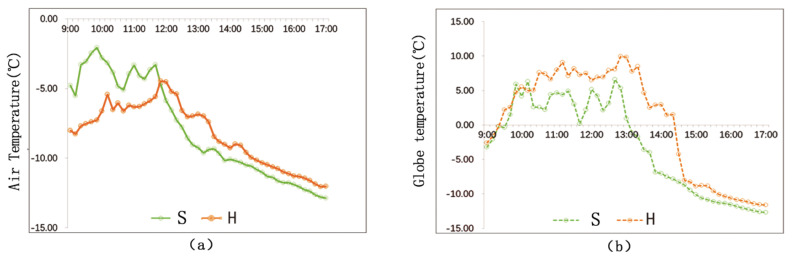
Temperature comparison at points S and H: (**a**) Ta; (**b**) Tg.

Figure 16a shows that the change trends of the air temperatures and the globe temperatures at the two points are similar. Before 12:00 p.m., the air temperature of S is higher than that of H. That is because the point S is enclosed on three sides, while point H is enclosed on two sides. The overall area walls influence the point S is larger. After 12:00 p.m., the air temperature of H is higher than that of S. The reason is that, with the change of solar azimuth, the walls near point S block sun radiation.

Figure 16b shows that the general globe temperature of H is higher than that of S. The buildings near S are covered by red glazed tiles, the radiation rate of which 0.69 (*ε* = 0.69). The buildings near H are covered by pink coating, the radiation rate of which is 0.95 (*ε* = 0.95). Generally speaking, smooth wall surface has stronger light reflectivity and lower radiation rate, while rough or dim wall surface has a comparatively weaker light reflectivity and higher radiation rate. Accordingly, walls at point H has stronger radiation than walls at point S. Thus, point H has a higher globe temperature. 

**Figure 17:**
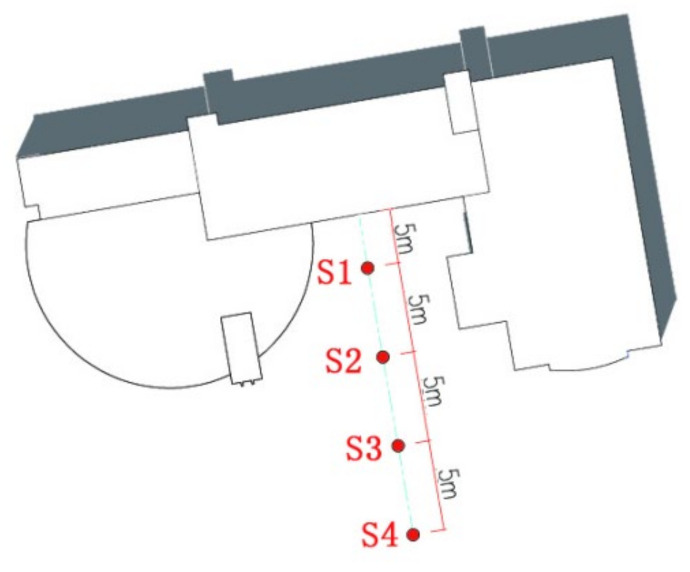
Setting of the measuring points near the building thermal interface.

To study the relationship between heat exchange of building walls and distance, we set four measuring points (S1, S2, S3, and S4) along the vertical direction of building interface S (see Figure 17). The distance between each measuring points is 5 m.

**Figure 18:**
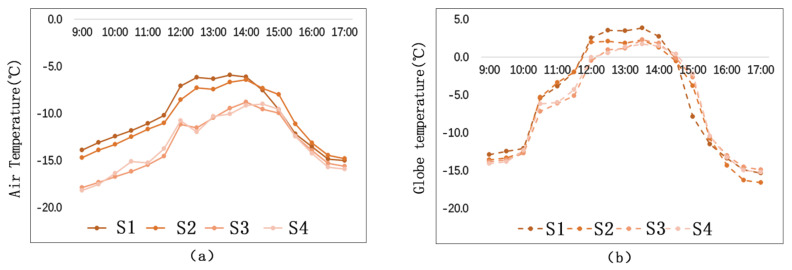
Temperature comparison among different measuring points of different distances: (**a**) Ta; (**b**) Tg.

Figure 18 shows that, on the vertical direction of the building interface, the air temperature and the globe temperature decrease with the increase of distance. When the distance exceeds 15 m, the building has little influence on the temperature. Before 2:30 p.m., the temperature differences among the measuring points are more obvious. While, after 2:30 p.m., the differences are not so obvious since the general temperature starts to decline quickly. The globe temperature differences among the measuring points are most obvious during 12:30 p.m.–2:00 p.m. In addition, the differences get smaller before and after this time period. 

**Figure 19:**
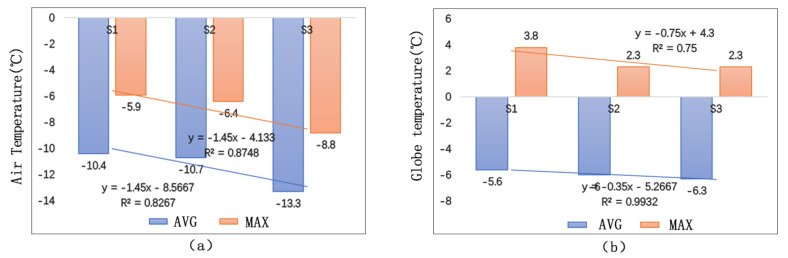
Temperature matching at different measuring points: (**a**) Ta Matching; (**b**) Tg Matching.

Figure 19 shows the matching relationship between temperature (including air temperature and globe temperature) and distance. The study demonstrates that air temperature has a closer relationship with the change of distance than globe temperature. From the perspective of thermal transfer, there are mainly two ways of thermal transfer between street walls and the surrounding environment: thermal convection and thermal radiation. The fact that air temperature is under a bigger influence of distance proves that thermal convection plays a more important role in rising outdoor temperature than thermal radiation.

### 3.3. Relationship between Street Morphology and Microclimate

SVF is the main factor influencing temperature in cities [35]. L/C reflects the closure condition of the environment. This study has measured air temperatures, wind speeds, PETs, SVFs, and L/Cs of six sites. Through linear regression analysis, the article studies the correlation between space morphology features and microclimate of streets.

#### 3.3.1. Correlation Analysis between SVF, L/C and Temperature

**Figure 20:**
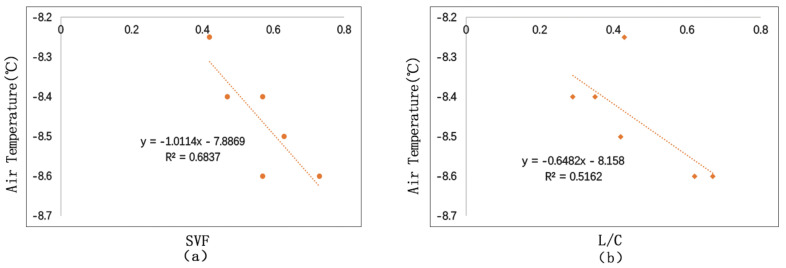
Correlation between SVF, L/C, and temperature: (**a**) correlation between SVF and Ta; (**b**) correlation between L/C and Ta.

As is shown in Figure 20, we conducted linear fitting between SVF, L/C and the average temperature of the day. Analysis shows that SVF and L/C are in negative correlation with air temperature. The reason is that winter is severely cold in these regions. Buildings with heating systems mainly serve as heating sources in the winter, which will increase the surrounding temperature to some extent. In addition, if the SVF and the L/C are small, wind field will have little effect on wind speed. This will also help to increase temperature. SVF and air temperature have a better degree of fitting, suggesting that their correlation is stronger. If the SVF increases 0.1, the air temperature will reduce 0.1 °C.

#### 3.3.2. Correlation Analysis between SVF, L/C, and Wind Speed

**Figure 21:**
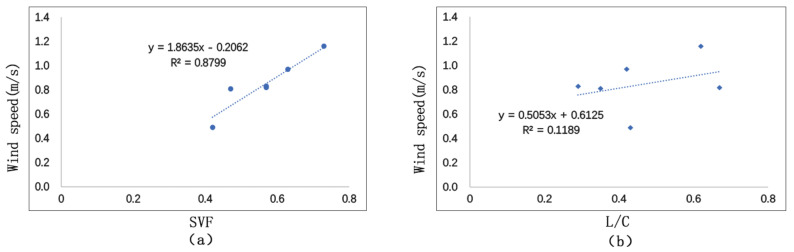
Correlation between SVF, L/C and wind speed: (**a**) correlation between SVF and wind speed; (**b**) correlation between L/C and wind speed.

Figure 21 shows the fitting situation of SVF and L/C with the average wind speed of the day. We could see that SVF and L/C are positively correlated with wind speed. Comparatively speaking, SVF has a closer relationship with wind speed than L/C. It also shows that wind speed is under the influence of surrounding environment. L/C and wind speed has a weak correlation (*R*^2^ = 0.1). This is because building forms have certain influence on wind field, which bring about vortex and blast. Generally, L/C is positively correlated with wind speed. The more open the space is, the faster the wind speed. If SVF increases 0.1, wind speed will increase 0.19 m/s.

#### 3.3.3. Correlation Analysis between SVF, L/C, and PET

**Figure 22:**
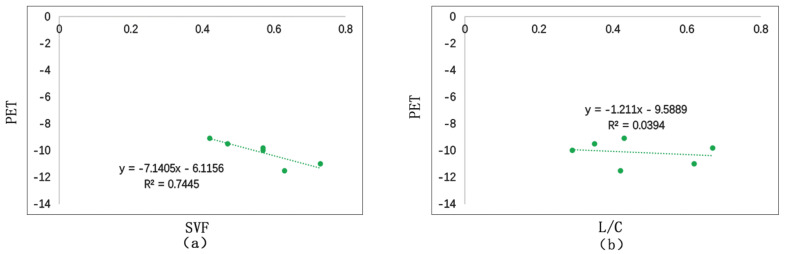
Correlation between SVF, L/C, and PET: (**a**) correlation between SVF and PET; (**b**) correlation between L/C and PET.

As is shown in Figure 22, we conduct linear fitting between SVF, L/C and the average PET of the day. The study demonstrates that PET is negatively related to SVF and L/C. That is to say, in cold regions, the more open the space is in winter, the lower the comfort level. L/C and PET have a lower degree of fitting (*R*^2^ = 0.03), suggesting that L/C has a weak correlation with PET. While SVF and PET have a higher degree of fitting, suggesting that SVF has a bigger influence on PET. If the SVF increases 0.1, the PET will reduce 0.7 °C.

## 4. Discussion

Currently, research on microclimate of urban blocks is mainly focused on air motion and temperature condition of street canyons. For example, previous research has studied the relationship between street canyon orientations and the amount of solar radiation received, the relationship between the height–width ratio of streets and flow field structure [36], and so on. The study objects are mostly urban streets, characterized by continuous and complete street interfaces. However, spatial features of college campus are different [23,24,25]. The common block morphology parameter (such as height–width ratio, street orientations) research on relationship thermal environment does not fully apply to the situation of campus streets.

According to our study, the deciduous trees along the streets can reduce wind speed in winter to some extent. The average wind speed can be reduced by 0.2 m/s, and the wind directions can be changed as well. The results are similar to Zhang’s research [37]. In addition, the wind speed differences of different sites are relatively big—the maximum difference reaches 1.1 m/s. SVF reflects the closure degree of outdoor space. SVF affects sunshine duration and wind speed [38]. This study finds that SVF of streets is negatively correlated with outdoor temperature in winter. This finding is different from the research results of previous scholars [21,22,23,24,25]. This is because building heating in winter serves as a heating source, and the temperature of its surrounding environment can be increased by the buildings to a certain extent. Therefore, if the SVF is small and the space is enclosed, the surrounding buildings will have a bigger influence on the surrounding environment. The study also finds that SVF of the streets is positively correlated with wind speed. This finding is consistent with Yang’s research [39]. However, given that the climate situations and the study objects of the two studies are different, the result of current study is slightly different from the previous one. SVF has a comparatively stronger influence on wind speed of the campus streets.

The study has its limitations. First of all, the space forms chosen for study are not inclusive. Secondly, the uncertain factors of the outdoor environment may affect the search results. In the follow-up studies, we will select more examples to enrich the search results. Furthermore, in our study of building interfaces, we have confirmed their influence degree and scope. We also studied the differences between two facings. However, we have not yet studied how other types of building facings and building volume will affect thermal environment. 

## 5. Conclusions

Through on-site measurement of the microclimate of a typical university campus in severe cold regions, this article studies the microclimate of different street types and the influence of building heating on the surrounding thermal environment. The conclusions are as follows:

### 5.1. Open-Type Streets

In winter, the trees alongside the streets block solar radiation to some extent. Thus, streets of different orientations have different temperatures. The study shows that street orientation has little influence on air temperature but has a more obvious impact on globe temperature. The average globe temperature of the EW-N5° oriented street is 1.3 °C higher than that of the SN-E15° oriented street. For open-type streets, the smaller the angle between the street orientation and the predominant wind direction, the greater the wind speed.

In winter, for streets of similar orientations, trees along the streets have little influence on air temperature but have a more obvious influence on globe temperature. The maximum globe temperature difference between streets with trees and streets without trees is 1.1 °C. With respect to wind speed, streets with trees can reduce wind speed (about 0.2 m/s less) and change wind directions.

### 5.2. Semi-Open-Type Streets

For semi-open-type streets, the locating direction of buildings is the main factor influencing microclimate. Buildings of different locations cause different degrees of sunshine blockage, thus resulting in different temperatures. The maximum air temperature difference of different streets is 2.1 °C. In addition, if the street buildings are located in similar directions, the temperature is related to closure degree of the streets. The higher the closure degree is, the higher the air temperature is. Wind speed is influenced by the relationship between buildings’ locating direction and predominant wind direction. If the building is located in the upwind part of the predominant wind direction, the wind speed is reduced. In addition, if the building interface is continuous and closed, its effect on reducing wind speed will be more obvious. When wind encounters building interface, a vortex will be formed, and the direction of wind can be changed.

### 5.3. Street-Entry-Type Streets

For street-entry-type streets, if the height–width ratios of the streets are similar, the continuity degree of the streets interface will be the main factor affecting streets microclimate. Build-to-line ratio is the parameter reflecting closure degree. The bigger the build-to-line ratio is, the space will be more closed, and more solar radiation will be blocked. 

### 5.4. Building Heating Interface

The building interface acts as a heating element and affects the surrounding thermal environment by heat convection and heat radiation. Analysis demonstrates that heat convection has a more obvious effect on rising temperature than heat radiation. Different building materials have different degrees of influence on the thermal environment. Pink coatings have a bigger influence on globe temperature than red glazed tiles because pink coatings have a bigger radiation rate. For streets enclosed by walls on three sides, temperature decreases with the increase of distance. However, the relationship between temperature and distance is not linear. When the distance exceeds 15 m, temperature will not change with the increase of distance. 

### 5.5. Street Morphology

SVF and L/C of streets are negatively related to temperature, and SVF has a bigger influence on temperature than L/C. If SVF increases 0.1, air temperature will reduce 0.1 °C. SVF and L/C are positively related to wind speed, and SVF has a closer correlation with wind speed. If SVF increases 0.1, wind speed will increase 0.19 m/s. SVF and L/C are negatively related to PET, and SVF has a stronger influence on PET than L/C. If SVF increases 0.1, PET will reduce 0.7 °C.

In China, the “Evaluation Criteria of Green Campus” currently in effect have not listed clear criteria for outdoor environment of campus. The “Evaluation Criteria” only stipulates certain criteria regarding wind speed and energy-saving ability of buildings.

The study of the streets’ microclimate on campus is a development for the general study of campus planning theories. For future design of campus in severe cold regions, designers should choose suitable street orientations based on its local climatic conditions. In addition, the overall arrangement can be designed to be relatively concentrated to reduce SVF and L/C. A more enclosed space will improve the environmental comfort level. If the campus in severe cold regions needs to be reorganized and expanded, we suggest that the designers should try to increase the space enclosure degree to improve human comfort by designing more buildings, scenery structures, or more trees on campus.

## Figures and Tables

**Table 1:** Basic meteorological information of Daqing (2007–2017).

Month	Daily Maximum Temperature (°C)	Daily Minimum Temperature (°C)	Mean Temperature (°C)	Relative Humidity (%)	Wind Speed (km/h)	Sunshine Duration
Annual	10	−1.3	3.6	65	12	2571.2
January	−13	−24	−19.4	72	17	155.9
February	−7	−20	−15.4	68	16	179.9
March	2	−10	−4.8	56	14	230.9
April	13	1	6.0	49	9	231.4
May	21	8	14.3	51	9	264.1
June	26	15	20.0	65	9	260.2
July	28	18	22.8	77	9	254.2
August	26	16	21.1	78	12	247.2
September	21	9	14.4	70	9	230.5
October	12	1	5.6	63	8	206.8
November	0	−10	−5.7	65	6	170.2
December	−9	−20	−15.6	71	8	139.9

**Table 2:** Test time and climatic conditions.

Date	Air Temperature	Wind Direction (Level)	Maximum Total Solar Radiation Intensity (W/m^2^ )
27 December 2018	−12–(−21) °C	Northwest Wind Level 2	555.1
14 January 2019	−6–(−17) °C	Northwest Wind Level 2	389.7
16 January 2019	−11–(−21) °C	Northeast wind level 2	535.8

**Table 3:** Instrument technical parameter.

Name and Model	Measuring Range	Accuracy	Sampling Period
BES-01 Temperature Recorder	Temperature: −30–50 °C	±0.5 °C	10 s to 24 h
Kestrel 4500 Small-Size Weather Station	Wind Speed: 0.4–40 m/s	±0.1 m/s	2 s to 12 h
	Temperature: −29 –(+70) °C	±1.0 °C	
	Wind Direction: 0°–360°	±5°	
	Relative Humidity: 5–95%	±3%	
	Air Pressure: 75–110 KPa (25 °C)	±0.5 kPa	

**Table 4:** Spatial features of the measuring sites.

Measuring Sites	Space Forms	Environment	Street Orientation	SVF	L/C	Angle to the Dominant Wind Direction	Build-to-Line Ratio
C1	Open type	No trees	EW-S10°	0.86	0.92	55°	
C2	Open type	Trees	EW-S5°	0.64	0.87	55°	
C3	Open type	Trees	SN-E15°	0.64	0.87	60°	
C4	Semi-open Type	Trees (multi)	SN-W2°	0.47	0.35	43°	
C5	Semi-open Type	Trees	SN-W27°	0.73	0.62	18°	
C6	Semi-open Type	Trees (multi)	SN-E36°	0.42	0.43	81°	
C7	Semi-open Type	Trees (multi)	SN-W42°	0.57	0.67	3°	
C8	Street-entry Type	No trees	SN-W9°	0.57	0.29	36°	32%
C9	Street-entry Type	No trees	SN-W9°	0.63	0.42	36°	62%

SVF, sky view factor; L/C **,** plane opening rate.
